# Inhibition of emotion-related autonomic arousal by skin pressure

**DOI:** 10.1186/s40064-015-1101-9

**Published:** 2015-06-26

**Authors:** Wataru Sato

**Affiliations:** Department of Neurodevelopmental Psychiatry, Habilitation and Rehabilitation, Graduate School of Medicine, Kyoto University, 54 Shogoin-Kawaharacho, Sakyo, Kyoto 606-8507 Japan

**Keywords:** Autonomic nervous system, Heart rate, Negative emotion, Skin potential response, Skin pressure reflex

## Abstract

**Background:**

Negative emotions can cause discomforting autonomic arousal, which can be difficult to inhibit using willpower alone. Although previous physiological studies have reported that skin pressure at certain bilateral locations reflexively inhibits sympathetic nervous system activity, few studies have tested the effect of this inhibition on emotion-related autonomic arousal in humans.

**Findings:**

I recorded skin potential response (SPR) and heart rate (HR) in healthy participants in response to loud noises presented concomitantly with or without skin pressure applied bilaterally to the sides of the chest. Weaker SPR and lower HR were observed in response to the noises accompanied by skin pressure.

**Conclusions:**

These findings indicate that skin pressure can be an easy and effective method to inhibit autonomic arousal related to negative emotions.

**Electronic supplementary material:**

The online version of this article (doi:10.1186/s40064-015-1101-9) contains supplementary material, which is available to authorized users.

## Background

Intense negative emotions, such as fear, elicit autonomic arousal, which can include cold sweat and rapid heartbeat. Although emotion-related autonomic arousal has been proposed as an evolutionarily acquired survival advantage (Porges 2009), it can also be a source of discomfort and have negative effects on behavior in modern humans (Williamson et al. 2013).

Inhibiting emotion-related autonomic arousal through willpower is quite difficult. Several mind–body intervention practices have been proposed, including muscle relaxation and deep breathing (for a review, see Kim et al. 2013). However, these practices usually require training for several weeks or more. Furthermore, empirical evidence has not consistently supported the efficacy of such practices on the activity of the autonomic nervous system (Kim et al. 2013). Developing easy and effective methods to inhibit autonomic arousal related to negative emotions would benefit all humans.

Previous physiological studies have reported that various species, including mice, cats, and humans, have a skin pressure reflex whereby mechanical pressure on certain bilateral epidermal areas reflexively inhibits the activity in the sympathetic nervous system (e.g., Takagi 1952; for a review, see Takagi 1975). The effects of skin pressure were exhibited in various types of autonomic measures, including skin potential response (SPR) and heart rate (HR) (Takagi 1952). The receptors of this reflex were found to lie in the skin organ (Takagi 1949). It was speculated that this reflex may have evolved to serve a basic biological function, such as a mother calming her children by biting their skin (Takagi 1975).

These data led to the hypothesis that the skin pressure reflex could suppress emotion-related autonomic arousal. Consistent with this idea, a previous study has anecdotally reported that cats do not exhibit emotional reactions in response to physical shocks to their tails while they are receiving skin pressure (Takagi 1975). However, few studies investigated the effects of skin pressure on emotion-related autonomic arousal in humans. One study described that SPRs in response to electrical shock appeared to be inhibited in humans as a result of skin pressure (Hayashi 1952). However, the study did not perform statistical analysis, and hence the evidence was not strong.

In the present study, I evaluated my hypothesis by recording SPR and HR, which reflect emotion-related autonomic arousal (Palomba et al. 2000), in response to loud noises, which are unconditioned stimuli that induce negative emotions (Damasio et al. 1990), in healthy participants presented concomitantly with or without bilateral skin pressure to the sides of the chest.

## Methods

### Participants

Twelve male volunteers (aged 19–29 years) participated in the experiment. All of the participants confirmed that they had normal hearing abilities and did not have any abnormal autonomic conditions such as those caused by the use of psychiatric medication. All participants gave informed consent to participate in this study, which was conducted in accordance with the ethical provisions of the Kyoto University Graduate School of Education and the Declaration of Helsinki.

### Equipment

A Synafit 360 polygraph system (San-ei, Tokyo, Japan) was used to continuously record SPR and HR. SPR was recorded using Ag/AgCl electrodes attached to the hypothenar eminence and forearm of the left hand. HR was recorded using a photoplethysmograph positioned on the last phalange of the left second finger with automatic online calculations of beats per minute. Data were filtered through a bandpass of 0.15–15 Hz and recorded on paper at 2 mm/s.

Skin pressure was applied using two handmade devices. The devices consisted of springs, which measured force, and 8-cm-wide square plates (i.e., 64 cm^2^ in area), which were flexible in order to fit the body surface.

Loud noises were created by using sports percussion caps (Evernew, Tokyo, Japan). A handmade remote control device was used to generate the noises. The device was placed 2.0 m behind participants. The sound level used was approximately 110 dB.

### Procedure

Experiments were conducted individually in an electrically shielded soundproof room. After the electrodes and photoplethysmograph were applied, each participant was seated comfortably in a chair in front of a white wall. Participants were requested to sit without leaning against the back of the chair; this position was selected because the skin of the back was found to be sensitive to the skin pressure reflex while the skin of the buttocks was not (Yamada 1950; see Additional file 1: Figure S1). Participants were given 10 min to adapt to the experiment room, and were then informed about the skin pressure and stimulus presentation. They were requested to relax and not to make body movements or breathe deeply. For practice, they received the skin pressure three times, for approximately 10 s each time, and were subjected to the noise once.

Skin pressure was applied from behind the participants by the experimenter using the pressure device. Pressure was applied bilaterally to the sides of the chest, centered at the points where the horizontal line that connects the nipples crosses the vertical lines that run downward from the armpits, which were found to be the most sensitive body parts for this skin pressure reflex (Yamada 1950). The amount of pressure was set at 10 kg/64 cm^2^, because previous studies have reported that the minimum intensity required to elicit this reflex is 3–6 kg/20 cm^2^ (Takagi 1954), that the reflex is more evident with more pressure and more surface area (Kawai 1954), and that the reflex disappears when the pressure causes subjective discomfort (Takagi 1975).

Four trials of noise presentation were conducted, consisting of two trials of each skin pressure conditions (pressure versus no pressure). Note that physiological responses to loud noises have been reported in as few as a single trial (e.g., Damasio et al. 1990; Lader and Wing 1964). In each trial, the noise was presented 10 s after an oral warning. In the skin pressure condition, pressure was applied simultaneously with the warning and continued for 25 s. Under the no-pressure condition, no pressure was applied. Intertrial intervals were 3 min. The two skin pressure conditions were presented one after the other, and the order was counterbalanced across participants.

### Data analysis

The data were analyzed using SPSS 10.0J (SPSS Japan). For SPR, peak-to-peak amplitude was calculated by subtracting the maximum negative deflection from the maximum positive deflection (cf. Hori et al. 1970; Knezevic and Bajada 1985) for 15 s after noise onset. HR change was calculated by subtracting mean HR during the 15 s after noise onset from those during the 10 s before noise onset. HR data from two participants were excluded from analysis because of equipment failure. SPR and HR data were averaged for each condition for each participant, and then analyzed by paired *t* tests. Preliminary analyses revealed that the HR did not differ significantly across the skin pressure vs. no-pressure conditions during the 10 s prior to noise onset [*M* ± *SE* = 70.0 ± 2.2 vs. 69.5 ± 2.4 beats per minute; *t*(9) = 0.62, *p* > .1], thereby supporting the validity of pre- vs. post-stimulus period subtraction; SPR peak-to-peak amplitude was significantly lower during the skin pressure condition compared to the no-pressure condition [*M* ± *SE* = 1.6 ± 0.5 vs. 3.5 ± 0.6 mV; *t*(11) = 2.54, *p* < .05; also discussed below].

## Results

**Figure 1:**
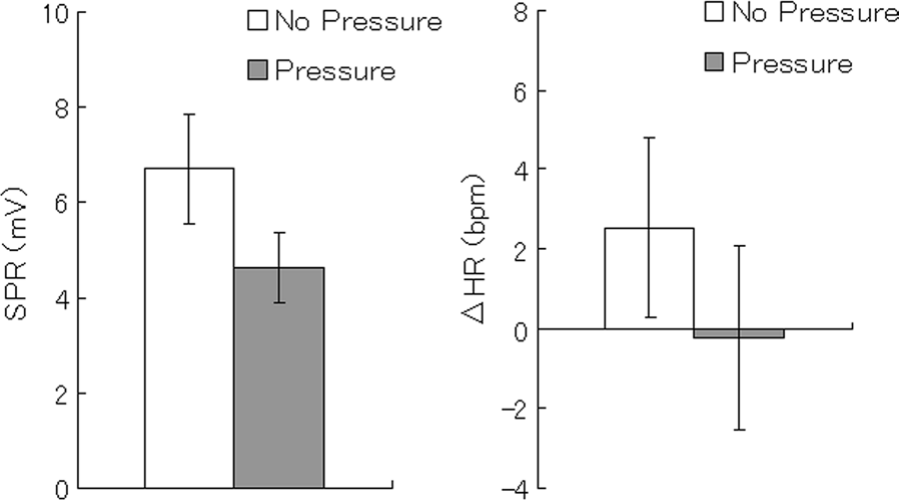
Mean (with *SE*) skin potential responses (SPRs) and heart rate changes (ΔHR) in beats per minute (bpm) in response to loud noises without and with skin pressure.

After exposure to the loud noise, SPR peak-to-peak amplitude (Figure 1, left) was significantly lower under the skin pressure condition than the no-pressure condition [*t*(11) = 2.69, *p* < .05]. HR change (Figure 1, right) was also significantly reduced under the skin pressure condition than the no-pressure condition [*t*(9) = 3.54, *p* < .01].

## Discussion

Both SPR and HR measures were reduced in response to loud noises under the bilateral skin pressure condition compared with the no-pressure. These results are consistent with previous evidence that bilateral skin pressure reflexively inhibits widespread sympathetic nervous activity (Takagi 1975). The results are also consistent with the finding that skin pressure modulates emotion-related autonomic arousal (Hayashi 1952). However, this previous investigation was descriptive. To my knowledge, this is the first statistically supported evidence that emotion-related autonomic arousal can be inhibited by bilateral skin pressure.

These results may have practical applications because emotion-related autonomic arousal can negatively influence human wellbeing, and can be reduced using skin pressure. Consistent with this idea, several researchers found similar phenomena through their experiences and utilized them to improve emotional states. For example, Grandin observed that cattle remained calm when receiving bilateral pressure to large areas while in a cattle chute, and later applied this technique to herself; she found that the application of skin pressure helped her to relax, and developed a device for humans (Grandin and Scariano 1986). She and her colleagues reported that the device reduced tension in normal adults (Grandin 1992) and anxious children with developmental disorders (Edelson et al. 1999). The current results provide physiological evidence to support the efficacy of such applications, and suggest that bilateral skin pressure may be an easy and effective method to inhibit emotion-related autonomic arousal.

Preliminary analysis showed that the SPR peak-to-peak amplitude was lower during the skin pressure condition compared to the no-pressure condition prior to noise onset. It should be noted that such a difference, occurring during the baseline period, would not change the interpretation of the inhibitory effect of skin pressure on emotional arousal, because the direction of the effect was opposite to the law of initial value. In which high pre-stimulus values generally induce small post-stimulus changes (Wilder 1962). It is possible that the warnings provided regarding the loud noises elicited anticipatory emotional responses (cf. Epstein and Clarke 1970), but the application of skin pressure inhibited these responses.

This study had several limitations that should be acknowledged. First, only a small sample of young males was recruited. Therefore, additional investigations using larger samples, and including participants of different genders and ages, are required to establish the effect of skin pressure on emotion-related autonomic arousal. Second, the comparison was conducted using only conditions of skin pressure and no-pressure, as in a previous study (Hayashi 1952). This design could be affected by confounding factors related to body contact (but not to the skin pressure reflex) such as attentional shift. Because a previous study reported that the effect of skin pressure differed according to body area in terms of resting-state autonomic activity (Yamada 1950), future studies should compare the effects of skin-pressure on responsive vs. non-responsive areas to clarify this issue. Finally, only loud noises were used to induce negative emotions, such that the generalizability of the skin-pressure effect to other types of emotional stimuli remains unproven. Future studies employing other types of emotional stimuli, such as photographs and scenarios depicting emotional events, could be used to confirm the autonomic-inhibitory effect of skin pressure related to negative emotions.

## Conclusions

In summary, SPR and HR were reduced in response to the loud noises under conditions of bilateral skin pressure relative to no-pressure. These results suggest that skin pressure can be an easy and effective method to inhibit autonomic arousal related to negative emotions.

## Additional file

Additional file 1:
**Figure S1.** The response-distribution of the skin pressure reflex throughout the whole body (Yamada 1950). The figure is used with permission from the publisher. +++ the most responsive; ++ highly responsive; + responsive; ± sometimes responsive; − not responsive.
