# Supplementary material for: Inhibition of emotion-related autonomic arousal by skin pressure
Source: Springerplus. 2015 Jun 26;4:294. doi: 10.1186/s40064-015-1101-9 (PMC4480267; doi:10.1186/s40064-015-1101-9)
Supplement: Additional file 1: — Figure S1. The response-distribution of the skin pressure reflex throughout the whole body (Yamada 1950). The figure is used with permission from the publisher. +++ the most responsive; ++ highly responsive; + responsive; ± sometimes responsive; − not responsive. [file 40064_2015_1101_MOESM1_ESM.pdf]

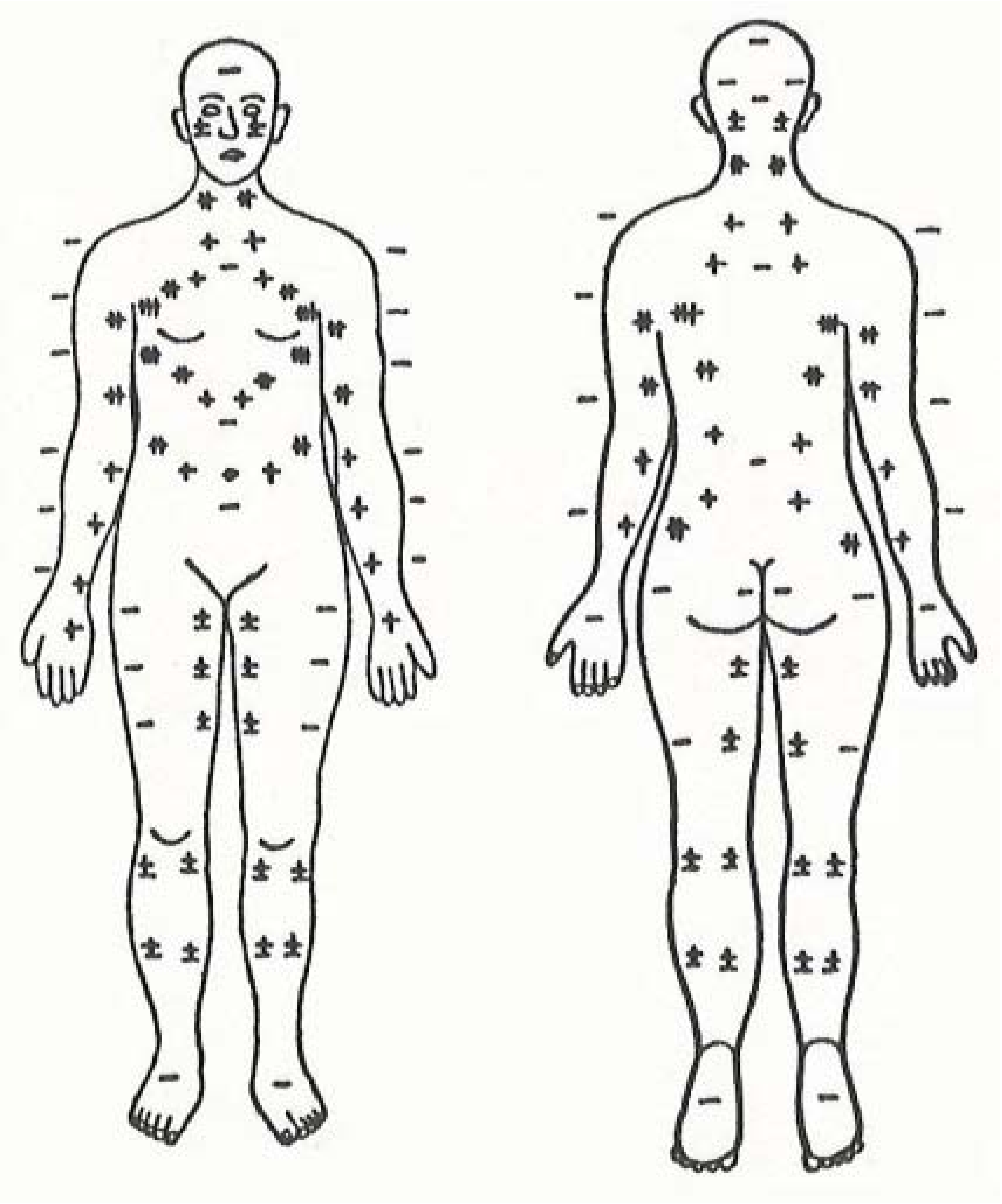

Figure S1. The response-distribution of the skin pressure reflex throughout the whole body (Yamada, 1950). The figure is used with permission from the publisher. +++ the most responsive; ++ highly responsive; + responsive; ± sometimes responsive; - not responsive.
